# Medicinal plants used by women from Agnalazaha littoral forest (Southeastern Madagascar)

**DOI:** 10.1186/1746-4269-9-73

**Published:** 2013-11-04

**Authors:** Mendrika Razafindraibe, Alyse R Kuhlman, Harison Rabarison, Vonjison Rakotoarimanana, Charlotte Rajeriarison, Nivo Rakotoarivelo, Tabita Randrianarivony, Fortunat Rakotoarivony, Reza Ludovic, Armand Randrianasolo, Rainer W Bussmann

**Affiliations:** 1 Department of Plant Biology and Ecology, Faculty of Science, University of Antananarivo, BP 566, Antananarivo 101, Madagascar; 2 Madagascar Research and Conservation Program, Missouri Botanical Garden, BP 3391, Antananarivo 101, Madagascar; 3 William L. Brown Center, Missouri Botanical Garden, PO Box 299, St. Louis, MO 63166-0299, USA

**Keywords:** Medicinal plants, Madagascar, Littoral forest, Traditional medicine, Women’s traditional knowledge

## Abstract

**Background:**

The country of Madagascar is renowned for its high level of biodiversity and endemism, as well as the overwhelming pressures and threats placed on the natural resources by a growing population and climate change. Traditional medicine plays an important role in the daily lives of the Malagasy for various reasons including limited access to healthcare, limited markets and traditional values. The objective of this study was to assess the modern utitilization of the Agnalazaha Forest by the local population in Mahabo-Mananivo, Madagascar, for medicinal plants used by women, and to establish a list of medicinal plants used by women sourced from Agnalazaha Forest.

**Methods:**

Ethnobotanical studies were conducted over a period of five months in 2010 to determine the diversity of medicinal plants used by women in the commune of Mahabo-Mananivo. In all, 498 people were interviewed, both male and female ranging age from 15 to over 60 years old.

**Results:**

152 medicinal plants used by local people were collected during the ethnobotanical studies. Among the recorded species, eight native species are widely used by women. These species are known for their therapeutic properties in treating placental apposition and complications during childbirth as well as tropical illnesses such as malaria, filariasis, and sexual diseases like gonorrhea and syphilis.

**Conclusions:**

Littoral forests are rare ecosystems that are highly threatened on the island nation of Madagascar. Our investigation into the use of medicinal plants sourced from and around the Agnalazaha Forest by the women of Mahabo-Mananivo reinforces the need for this natural resource as a first line of health care for rural families.

## Background

Traditional medicine is a term used to describe the use of natural resources, often in concert with ritual and spirituality, to prevent, treat and heal human diseases and ailments [1]. While the use of plant species for healing dates back further than the written record, with evidence the Neanderthals practiced plant medicine [2], it is still being used by many in our modern era. Eighty (80) percent of the world's population depends on traditional medicine for the treatment of pain [3]. And in developing countries such as Madagascar medicinal plants remain a primary source of medical care [4] especially in very remote areas or in case of limited health resources.

Medicinal plant use in Madagascar has the added concern of biodiversity loss, environmental degradation, and sustainability. The island nation of Madagascar separated from Africa some 170 million years and the Indian subcontinent nearly 88 million years ago and the isolated flora and fauna have evolved with a high degree of microendemism [5]. Current floristic calculations indicate Madagascar houses between 12,000 and 14,000 vascular plant species, of which 90% are endemic [6] and 96% endemism in tree species [7]. However, the increasingly intense population growth has led to rapid deforestation as land is cleared for agricultural fields and for fuel [8]. Biodiversity loss, in general, has severe implications on environmental stability which in turn affects human health [9]. When biodiversity directly adds to the wellness of a community as a resource for medicine, biodiversity loss can have even deeper consequences as medicinal plant species are lost or are no longer available [10,11].

Within Madagascar, one of the most threatened ecosystems is the littoral forest [12]. Although the littoral forests of Madagascar once stretched 1600-km along the eastern coast as one single biological corridor, there is only 10% of the original forest remaining [13]. One such littoral forest, the Agnalazaha Forest, is located in the rural commune of Mahabo-Mananivo, 750 km southeast of the capitol city of Antananarivo. Approximately 72.3% of the flora of Agnalazaha is endemic to Madagascar [14].

The villages of Mahabo-Mananivo source timber and non-timber forest products from Agnalazaha Forest littoral forest. Furthermore, the community of Mahabo-Mananivo still practice and often prefers traditional medicine, especially for common diseases and infectious diseases [15]. As is the case with most familial systems, the first line of healthcare decisions and action is often administered by female household members [16]. The purpose of this study was to assess the modern utilization of this forest by the local population with a focus on the plants known and utilized by women in their everyday care giving. We focused on the women for this study while a study on the use of medicinal plants by men was carried out simultaneously. At times men were present during the interview process and would add information about plants used by women which we allowed.

## Methods

Research was coordinated by and supported in large part by the staff at the Missouri Botanical Garden Mahabo-Mananivo Conservation research site. Field research was conducted over a period of five months (January – May) in 2010 with three field trips to the community. A ten day preliminary exploration was used to become familiar with the community and introduce ourselves, make contact with local officials and present the topic of our research. A hired local guide acted as our translator, introduced us to interview prospects and coordinated interview schedules. Consent was given by the tribal leaders, local government officials and by each individual we interviewed.

### Study site

**Figure 1:**
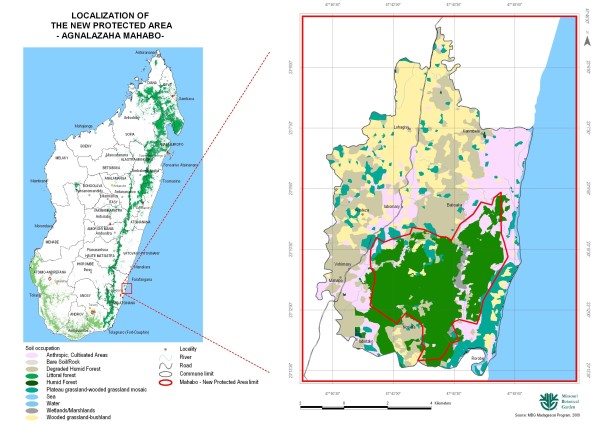
**Map of study area.** Location of Mahabo-Mananivo community within context of Madagascar and in relation to Agnalazaha Forest.

Agnalazaha Forest is located within the district of Farafangana, Atsimo Atsinanana region in southeastern Madagascar, in the Commune Rural Mahabo-Mananivo (Figure 1). The National Road 12, a paved highway connecting Farafangana and Vangaindrano borders the forest to the west while the Indian Ocean borders it to the east. It is between 47° 41′and 47° 45′ E, and 23° 09′and 23° 14′ S with an altitude of less than 50 m [14]. In 2003, it was measured that this coastal forest covered an area of 1,565 ha and represents approximately 17% land coverage of the rural area of the commune Mahabo-Mananivo. Agnalazaha Forest has the status of Forest Reserve under article number 129-SF/EF/CG since May 17, 1954, but has been under the management of the Missouri Botanical Garden (MBG) since 2002.

The southeast region of Madagascar is characterized as the eastern coastal plain and has a climate of high rainfall and high average temperature [17]. The Agnalazaha Forest experiences two seasons: the hot rainy season from December to April, and the cool season from May to November. The average annual rainfall in Agnalazaha Forest is 2,706 mm. The average annual temperature varies between 21°C - 24°C (69 °F – 75 °F). According to the bioclimatic division of Madagascar, this region belongs to the humid tropics and part of the humid warm bioclimatic type [18]. Agnalazaha Forest is classifed as a littoral forest, characterized by an open canopy and sandy soils [12], seasonally flooded wooded swamps, open marshes with *Nepenthes madagascariensis* and *Lepironia articulata*, savannas, remnants of secondary forest on lateritic soils and reforestation forests of *Eucalyptus robusta* and *Acacia mangeum*. There are 275 species of plants in Agnalazaha Forest [14] distributed within 188 genera and 82 families. The site contains species belonging to three endemic families, Asteropeiaceae (2 species), Sarcolaenaceae (6 species) and Sphaerosepalaceae (1 species). Furthermore, 199 species present in Agnalazaha Forest are determined to be endemic to Madagascar (72.3%).

An inventory of primates [19] conducted in Agnalazaha Forest identified four species of lemur all of which are considered to at least be threatened, including the critically endangered *Eulemur albocollaris*[20]. All are known to be hunted locally. A similar study identified seven species of endemic small mammals found in Agnalazaha Forest including *Pteropus rufus, Hemicentetes semispinosus, Setifer setosus and Tenrec caudatus, Hova oryzorictes*, all of which are also locally hunted [21].

### Surrounding community

Commune Rural Mahabo-Mananivo surrounds Agnalazaha Forest to the west, north and south. There are 6,998 residents according to the 2009 census. Mahabo-Mananivo is primarily comprised of residents identifying with the Antesaka ethnic group, while Antefasy, Merina and Betsileo members have migrated to this area as well. The municipality of Mahabo-Mananivo consists of ten fokontany surrounding the Agnalazaha Forest; Mahabo, Vohimasy, Iabotako, Nosiala, Iambomary, Baboaka, Lohagisy, Karimbelo, Rorobe, and Agnateza. A “fokontany” is the smallest political distinction recognized by the government. It may compromise several small villages with an average 1,000 people [22]. Mahabo-Manaivo is primarily an agriculture economy. Approximately 99.74% of the population is farmers. Rice fields dominate the landscape with cassava, yams, and manihot as supplementary crops. Additional income is sought through handicraft production, largely basketry weaving. The most popular species used for basketry is *Lepironia articulate*, *Cyperus sp*, and *Pandanus sp*. Monthly income is less than 80,000 Ariary (approximately USD$35) for a majority of the population of Mahabo-Mananivo.

There is a relatively new community health center, built in 2009, in the fokontany Mahabo, located on Road 12. It has 2 rooms and accommodates one doctor and one nurse. However, a majority of the population of Mahabo-Mananivo relies on medicinal plants to cure common diseases. To this end, local people consult traditional healers called *ombiasy* to be treated with medicinal plants. *Ombiasy* can be distinguished into four different types of healers: *tromba* (spiritually possesed) healers, midwives, massage healers and premonition healers.

### Ethnobotanical surveys

The Agnalazaha Forest provides the local population with firewood, timber for home construction, non-timber products and medicinal plants. In order to identify medicinal plants known to be used by and for women in the rural commune of Mahabo-Mananivo, inquiries on the therapeutic use of plants were conducted primarily with women and female healers, although some men were interviewed as well. Due to time limitations, not all fokontany were included in the study. Fokontany were selected using the following criteria: (a) proximity to Agnalazaha Forest (b) Distance to the health center located in Mahabo (c) presence of female healers in the village. Fokontany closest to Agnalazaha Forest were given priority. Field visits to each fokontany selected were scheduled so that the villages furthest from the forest were visited first. The interviews were structured as semi-direct interviews with open questions [23]. The interviews were conducted with both individuals and in group settings [24]. Interview questions were written with two different approaches, inquiry of plant specific use or through disease-specific and/or symptomatic description plant use. Questionnaires or survey forms were established, first on medicinal plants used by women and healers, then the socio-economic and cultural value for each species (Additional file 1).

Surveys focused on plants used in the treatment of common female diseases in the commune. They were conducted with traditional healers (*ombiasy*), birth attendants, women and men who know the medicinal plants used by and for women. The interviews were interspersed with forest walks with interview participants where species were identified by their vernacular names and photos were taken. Herbarium voucher were made and the identification of these species was then conducted in the national herbarium of Tsimbazaza (TAN).

## Results

### Demographic variables

In the community 498 people were surveyed, 301 (60.44%) were women and 197 (39.56%) were men and 90.56% of those interviewed responded that they utilize medicinal plants. Table 1 compares the number of those who utilize medicinal plants with those who do not use medicinal plants for each age group, level of schooling, marital status and income level.

**Table 1:** Demographic information of the ethnobotanical interviewees

		**Number of people interviewed**	**Percentage of total**	**Utilize medicinal plants (#)**	**Percentage of total**	**Do not utilize medicinal plants (#)**	**Percentage of total**
**Gender**	Females	301	60.44	280	93.02	21	6.98
	Men	197	39.56	171	86.8	26	13.2
**Age group**	[15-19]	35	7.03	16	45.73	19	54.27
	[20–29]	84	16.87	73	86.91	11	13.09
	[30–39]	111	22.29	105	94.59	6	5.41
	[40–49]	117	23.49	115	98.29	2	1.71
	[50–59]	104	20.88	100	96.15	4	3.85
	[60 +]	47	9.44	42	89.36	5	10.64
**Level of**	Illiterate	328	65.9	316	96.34	12	3.66
**Education**	Primary	143	28.7	118	82.52	25	17.48
	Secondary	24	4.8	16	66.53	8	33.47
	University	3	0.6	1	33.33	2	66.67
**Marital status**	Single	105	21.1	80	76.21	25	23.79
	married	384	77.1	368	95.83	16	4.17
	widowed	9	1.8	3	33.07	6	66.93
**Household**	<80,000	371	74.5	349	94.07	22	5.93
**Education(monthly income in Ariary)**	80,000-160,000	123	24.7	99	80.49	24	19.51
	>160,000	4	0.8	3	74.9	1	25.1

Comparison of age group, level of schooling, marital status and income level of the 498 interview respondents of Mahabo-Mananivo.

People aged 40 to 49 years have the highest frequency of use of medicinal plants at 98.29%. This age group was followed by the 50 – 59 year old age bracket (96.15%), the 30 – 39 year old age bracket (94.59%), 60 years and older bracket (89.36%), the 20 – 29 years old bracket (86.91%) and finally the youngest bracket, 15 – 19 years old at 45.73%. We found that people at least 30 years old have increased knowledge in terms of medicinal plants, while lower knowledge levels occur in the younger age groups.

Furthermore, the data analysis shows that in the Commune of Mahabo-Mananavio, the majority of women (65.90%) who use were interviewed are illiterate, with 96.34% of them using medicinal plants. This high percentage is directly correlated with the fact that girls receive less education than boys. Persons with at least a primary school level of education made up 28% of our interviewees, and have a significant percentage of use of medicinal plants (82.52%), while those with secondary level of education (4.8% of our respondents) make little use of medicinal plants (66.53%). This percentage decreases again and becomes less significant for those with a university level education (33%).

Married people have a broad knowledge of medicinal plants with a percentage of 77.10%, while persons listed as single use plants at a frequency of 21.10%. Most of these respondents are single mothers who prefer to practice traditional care during childbirth and/or childhood diseases.

### Diversity of medicinal plants and their application

152 medicinal plants were recorded during our ethnobotanical interviews as part of the collective women’s pharmacopeia. The diversity of medicinal plants in the botanical groups shows that dicotyledons have a very high percentage of use (87%), followed by 8% of monocotyledons and finally 5% of pteridophytes. The most important medicinal families are: Asteraceae (11 species), Poaceae and (9 species), Myrtaceae, Euphorbiaceae and Fabaceae (6 species each), Rubiaceae (5 species), Apocynaceae and Zingiberaceae (4 species each), Anacardiaceae, Moraceae, Melastomataceae and Solanaceae (3 species each). Our findings illustrate the most well known and cited species by women have a high rate of endemism or regional nativity. (Table 2).

**Table 2:** Species known to be medicinal by women in Mahabo-Mananivo

**Family**	**Scientific name**	**Vernacular name**	**Part used**	**Disease treated**	**Distribution[**[6]**,**[25]**]**
AMARANTHACEAE	Amaranthus sp	Anampatsa	bark	intestinal parasites	
AMARANTHACEAE	*Chenopodium ambrosioides* L.	Taimboritsiloza	Entire plant	Placental apposition - Parasites - Nosebleeds	Naturalized in Madagascar
ANACARDIACEAE	*Mangifera indica* L.	Manga	Bark Root	Evacuation of the placenta - Diarrhea - Hemorrhoid - Leucorrhoea - Dental Disease - Gonorrhea	Naturalized in Madagascar
ANACARDIACEAE	*Rhus taratana* (Baker) H. Perrier	Taranta	Leaf	Poisoning - Convulsions - Epilepsy - Stomach pain	Endemic to Madagascar
ANACARDIACEAE	*Sclerocarya birrea* (A. Rich.) Hochst.	Sakoa	Leaf	Venereal diseases - Sedative - Astringent - Spider Bite	Comoros, Africa
ANNONACEAE	*Annona reticulata* L.	Coeur de Boeuf	Leaf	Evacuation of the placenta	
ANNONACEAE	Annona sp.	Sarisoky	Leaf	Stomach pain	
APHLOIACEAE	*Aphloia theiformis* (Vahl) Benn.	Fandramana	Leaf, Bark	Evacuation of the placenta - Malaria - Tuberculosis - Sore throat - Heartburn	Comoros, Mascarenes, Seychelles, Africa
APOCYNACEAE	*Catharanthus roseus* (L.) G. Don	Vonenina	Entire plant, Root	Stomach pain - Pancreas pain - Cancer	Endemic to Madagascar
APOCYNACEAE	*Petchia erythrocarpa* (Vatke) Leeuwenb.	Hentona	Bark	Malaria	Comoros
APOCYNACEAE	*Petchia madagascariensis* (A. DC.) Leeuwenb.	Kabokala	Leaf	Insect bites	Endemic to Madagascar
APOCYNACEAE	*Voacanga thouarsii* Roem. & Schult.	Kaboky	Leaf- Latex - Roots - Bark-seeds	Evacuation of the placenta - Hypertension - Heart problems-wounds - Boils - Gonorrhea-Eczema - Scabies - Fungal Infections - Rheumatism - Stomach pain	Africa
AQUIFOLIACEAE	*Ilex mitis* (L.) Radlk.	Hazondrano	Leaf	Bad luck	Africa
ARACEAE	*Colocasia esculenta* (L.) Schott	Saonjo	Leaf	Evacuation of the placenta	Naturalized in Madagascar
ARACEAE	*Typhonodorum lindleyanum* Schott	Via	Leaf, heart	Evacuation of the placenta - Burn - hip problems	Comoros, Mascarenes, Africa
ARALIACEAE	*Schefflera longipedicellata* (Lecomte) Bernardi	Membolo - vatsila		Epilepsy - Cold - Gonorrhea	Endemic to Madagascar
ARALIACEAE	*Schefflera sp.*	Memboloha	Leaf	Albumin - Worms - Plague - Evacuation of placenta	
ASCLEPIADACEAE	*Pentopetia* sp	Tandrokosy	Leaf, Stem	Eye disease - Jaundice - Gonorrhea	
ASPARAGACEAE	*Dracaena reflexa* Lam.	Hasina	Leaf - stem	Evacuation of the placenta - Malaria - Epilepsy	Mascarenes, Africa
ASPARAGACEAE	*Dracaena reflexa* var. *cernua* (Jacq.) Baker	Fananaraha	Leaf - stem	Placental apposition - Thinning	
ASPLENIACEAE	*Asplenium sp.*	Apanga malemy	Entire plant	gonorrhea	
ASTERACEAE	*Acanthospermum hispidum* DC.	Bakakely	Leaf	Diarrhea	Africa
ASTERACEAE	*Ageratum conyzoides* L.	Ananjazavavy	flowers	Stomach pain	Naturalized in Madagascar
ASTERACEAE	*Emilia sp.*	Kitsitsona	Leaf	Eczema - Ulcer	
ASTERACEAE	*Emilia* sp.	Tsiotsio	Leaf	Apposition of the placenta	
ASTERACEAE	*Helichrysum sp.*	Aferombohitra	Leaf	Scabies	
ASTERACEAE	*Mimosa pudica* L.	Ramoria	Leaf	Hepatitis - Albumin	Naturalized in Madagascar
ASTERACEAE	*Psiadia altissima* (DC.) Drake	Dinga	Leaf	Wounds	Endemic to Madagascar
ASTERACEAE	*Sigesbeckia orientalis* L.	Tsindaory	Leaf	Wounds	Naturalized in Madagascar
ASTERACEAE	*Vernonia appendiculata* Less.	Asotry	Leaf	tooth decay	Endemic to Madagascar
ASTERACEAE	*Vernonia exserta* Baker	Seva	Leaf	Chickenpox - Parasites	Endemic to Madagascar
ASTERACEAE	*Vernoniopsis caudata* (Drake) Humbert	Maranitry atoraky	Bark	Chickenpox	Endemic to Madagascar
ASTEROPEIACEAE	*Asteropeia micraster* Hallier f.	Manoky mena	Bark, Leaf	Evacuation of the placenta - Diarrhea - Fatigue - Mumps	Endemic to Madagascar
ASTEROPEIACEAE	*Asteropeia multiflora* Thouars	Manoky fotsy	Leaf Bark	Evacuation of the placenta - Malaria - Parasites - Dental Disease - Gonorrhea Fatigue	Endemic to Madagascar
BIGNONIACEAE	*Phyllarthron madagascariense* K. Schum.	Resiriky/ Zahana	Leaf	Malaria - Breastfeeding-Cough - disease of the hip	Endemic to Madagascar
BROMELIACEAE	*Ananas comosus* (L.) Merr.	Mananasy		Intestinal parasites - diarrhea	Tropics
BURSERACEAE	*Protium sp*	Ambihitry	Bark	Abscess - poisoning	
CALOHPYLLACEAE	*Calophyllum inophyllum* L.	Vintanina		trigeminal neuralgia	Comoros, Africa, New World
CANELLACEAE	*Cinnamosma fragrans* Baill.	Kanely	Bark	Cold - intestinal parasite - Headaches - Against poison	Endemic to Madagascar
CANELLACEAE	*Cinnamosma madagascariensis* Danguy	Fotsinana	Bark, leaf	Evacuation of the placenta - Malaria - Hepatitis - Epilepsy - Intoxication - Dysentery - Carrie dental	Endemic to Madagascar
CANNABACEAE	*Cannabis sativa* L.	Rongony	Leaf	Liver disease	
CARICACEAE	*Carica papaya* L.	Paza	Leaf, Fruit, seeds, roots	Breastfeeding - Headaches - Wounds - Menstrual Pain - Stomach: Ulcer Constipation - Indigestion - Boil - Cysticercosis - Toxoplasmosis - Cough - Yellow Fever - Tooth Decay	Tropics
COMBRETACEAE	*Terminalia catappa* L.	Atafa	Leaf	Ovarian cycle disruption - Albumin - Tension - Stomach pain	Madagascar, Comoros, Seychelles, Africa
CONNARACEAE	*Agelaea pentagyna* (Lam.) Baill.	Rangahtsara	Bark	Gonorrhea - Aphrodisiac - Stomach ache	Comoros, Mascarenes, Africa
CONVOLVULACEAE	*Ipomoea batatas* (L.) Lam.	Vomanga	Leaf	Pregnancy - Evacuation of the placenta - Insect stings	Naturalized in Madagascar
CRASSULACEAE	*Kalanchoe prolifera* (Bowie ex Hook.) Hamet	Silafafa	Leaf	Asthma - Cough - Rheumatism	Endemic to Madagascar
CUCURBITACEAE	*Cucurbita maxima* Duchesne	Voatavo	Leaf	Fever - colic	Naturalized in Madagascar
CYPERACEAE	*Cyperus papyrus* subsp. *madagascariensis* (Willd.) Kük.	Zozoro		Difficulty after childbirth - painful spasms	
CYPERACEAE	*Pycreus mundtii* Cherm.	Ahibita	Entire plant	Evacuation of placenta-Malaria - Tuberculosis	Mascarenes, Africa, New World
EBENACEAE	*Diospyros sp*	Hazominty	Leaf	Malaria	
ERICACEAE	*Agauria salicifolia* (Comm. ex Lam.) Hook. f. ex Oliv.	Haronga-panihy	Leaf	Scabies (Adult) - Wounds - Ulcers	Mascarenes, Africa
ERICACEAE	*Erica sp.*	Anjavidy	Leaf Stem leaves	Evacuation of placenta-Pneumonia - Syphilis	
ERYTHROXYLACEAE	*Erythroxylum ferrugineum* Cav.	Menahihy	Bark Leaf	Evacuation of the placenta - Diarrhea - Anemia	Endemic to Madagascar
ERYTHROXYLACEAE	*Erythroxylum gerrardii* Baker	Fanjoana	Leaf Bark	Yellow fever - Epilepsy	Africa
EUPHORBIACEAE	*Croton noronhae* Baill.	Tsiavadika	Bark-Leaf	Placental apposition - Malaria - Cough	Endemic to Madagascar
EUPHORBIACEAE	*Euphorbia hirta* L.	Jean Robert	Entire plant	Gonorrhea - Dysentery - Albumin	Naturalized in Madagascar
EUPHORBIACEAE	*Jatropha curcas* L.	Savoa	Leaf Latex	Evacuation of placenta Asthma - Dental Disease - Pneumonia	Naturalized in Madagascar
EUPHORBIACEAE	*Macaranga oblongifolia* Baill.	Mokarana	Leaf	Malaria - Diarrhea	Endemic to Madagascar
EUPHORBIACEAE	*Macaranga sp*	Mokarana	Leaf	Diarrhea	
EUPHORBIACEAE	*Manihot utilissima* Pohl	Kazaha	Leaf	Gonorrhea - painful spasms - Pneumonia - Boil	
EUPHORBIACEAE	*Suregada boiviniana* Baill.	Lelangana	Leaf	Placental apposition - Dysentery - Epilepsy-Malaria	Endemic to Madagascar
FABACEAE	*Albizia gummifera* (J.F. Gmel.) C.A. Sm.	Volomborona	Leaf	Fatigue - Cough	Africa
FABACEAE	*Cajanus cajan* (L.) Huth	Ambatry	Leaf	Evacuation of the placenta - Tension	Naturalized in Madagascar
FABACEAE	*Chamaecrista mimosoides* (L.) Greene	Quatre épingles	Leaf	Thrush - Schistosomiasis	
FABACEAE	*Intsia bijuga* (Colebr.) Kuntze	Hintsy	Leaf	Placental apposition - Cough	Mascarenes, Africa
FABACEAE	*Mimosa pudica* L.	Ramoria		Pelvic pain - Nervousness - Diuretic	Naturalized in Madagascar
FABACEAE	*Senna alata* (L.) Roxb.	Quatre épingles	Leaf	Hypertension	Naturalized in Madagascar
GENTIANACEAE	*Tachiadenus carinatus* (Desr.) Griseb.	Malanilava	Entire plant	Diarrhea	Endemic to Madagascar
GLEICHENIACEAE	*Sticherus flagellaris* (Bory ex Willd.) Ching	Ringotra	Leaf	Diarrhea - Measles - Vomiting - Coughing	Mascarenes, Comoros
HYPERICACEAE	*Harungana madagascariensis* Lam. ex Poir.	Harongana	Bud Leaf	Gonorrhea - heart disease - Albumin - Asthma - Boil-Diarrhea	Comoros, Mascarenes, Africa
ICACINACEAE	*Cassinopsis madagascariensis* Baill.	Valotry	Leaf - Bark	Cough - Itching - Syphilis	Endemic to Madagascar
LAMIACEAE	*Ocimum gratissimum* L.	Romba be	Leaf	Placental apposition - Asthma - Albumin - Headache - Dental Disease	Comoros, Mascarenes, Seychelles, Africa, Asia
LAMIACEAE	*Salvia coccinea* Buc’hoz ex Etl.	Romba madinika	Leaf	parasites	Naturalized in Madagascar
LAURACEAE	*Persea americana* Mill.	Zavoka	Leaf	Diarrhea - Apposition of placental - Cough	Naturalized in Madagascar
LECYTHIDACEAE	*Barringtonia racemosa* (L.) Spreng.	Fotatry	Leaf	Placental apposition - Scabies - Tetanus	Comoros, Australiasia, Africa
LILIACEAE	*Asparagus simulans* Baker	Ahitsifantatry	Entire plant	Epilepsy - Stomach pain	Endemic to Madagascar
LOMARIOPSIDACEAE	*Nephrolepis cordifolia* (L.) C. Presl	Mitsisiloha	Entire plant	Malaria	Mascarenes, Australasia, Seychelle, Afria, Asia, New World
LORANTHACEAE	*Bakerella sp*	Velomiato	Entire plant	Convulsion - Cough - Boil	
LYCOPODIACEAE	*Lycopodiella cernua* (L.) Pic. Serm.	Tongotsokina	Entire plant	Asthma - Epilepsy - Pelvic Pain - Gonorrhea - Cough - Hypertension	Mascarenes, Africa, Asia, New World
LYCOPODIACEAE	*Lycopodium clavatum* L.	Dito	Leaf	Pregnant - Placental apposition - Gonorrhea - Filariasis - Malaria	Comoros, Mascarenes, Africa
MELASTOMACEAE	*Clidemia hirta* (L.) D. Don	Voatrotrokala	Leaf	Wounds	Naturalized in Madagascar
MELASTOMACEAE	*Dichaetanthera sp*	Felabarika	Leaf	diarrhea	
MELASTOMACEAE	*Medinilla*	Takasina		Cough	
MELIACEAE	*Melia azedarach* L.	Voandelaka	Leaf	Fatigue	Naturalized in Madagascar
MENIPERMACEAE	*Burasaia australis* Scott-Elliot	Sompatry	Leaf	Intoxication - Convulsion - Dental Disease - Malaria - Medicinal plant magic	Endemic to Madagascar
MOLLUGINACEAE	*Mollugo nudicaulis* Lam.	Aferotany	Entire plant	Malaria - Albumin - Convulsion - Cough - Diarrhea - Diarrhea - Blood loss - Scabies	Australasia, Africa, New World
MONIMIACEAE	*Tambourissa castri-delphinii* Cavaco	Amborabe	Leaf	Placental apposition - Dysentery	Endemic to Madagascar
MONIMIACEAE	*Tambourissa parvifolia* Baker	Ambora	Leaf	Filariasis - Loss of blood	Endemic to Madagascar
MORACEAE	*Artocarpus altilis* (Parkinson) Fosberg	Soanambo	Leaf	Diarrhea	
MORACEAE	*Ficus polita* subsp. *polita*	Mandresy	Leaf	Placental apposition - bilious - Gonorrhea - Syphilis	
MORACEAE	*Ficus reflexa* Thunb.	Laza	Leaf	Pelvic pain - Gonorrhea	Comoros, Mascarenes, Seychelles
MUSACEAE	*Musa × paradisiaca* L.	Akondro	Leaf - fruit	Placental apposition - Diabetes - Prevents tooth decay - Diarrhea - Wounds	Naturalized in Madagascar
MYRICACEAE	*Morella spathulata* (Mirb.) Verdc. & Polhill	Hazosiay	Leaf	Placental apposition - Malaria - Cough - Stomach Pain - Dental Disease- Injury	Africa
MYRISTICACEAE	*Brochoneura acuminata* (Lam.) Warb.	Raraha	Leaf	Injury - Scabies - Abscess	Endemic to Madagascar
MYRTACEAE	*Melaleuca*	Kininy bonaky	Leaf	Placental apposition - Cold	Naturalized in Madagascar
MYRTACEAE	*Psidium cattleyanum* Sabine	Angavombazaha	Leaf	Diarrhea	Naturalized in Madagascar
MYRTACEAE	*Psidium guajava* L.	Angavogasy	Leaf	Malaria - Colic stomach - diarrhea - dysentery	Naturalized in Madagascar
MYRTACEAE	*Psidium guajava* L.	Angavofotsy	Leafs Roots	Diarrhea - Vomiting Boil	Naturalized in Madagascar
MYRTACEAE	*Syzygium aromaticum* (L.) Merr. & L.M. Perry	Jirofo	Leaf	Placental apposition - Dental Disease - Malaria	
MYRTACEAE	*Syzygium bernieri* (Drake) Labat & G.E. Schatz	Rotry	Bark-Leaf	Placental apposition - diarrhea - Dentistry Diseases - Scabies	Endemic to Madagascar
MYRTACEAE	*Syzygium emirnense* (Baker) Labat & G.E. Schatz	Rotry	Bark-Leaf	Placental apposition - Dentistry Diseases - Scabies	Mascarenes
NEPENTHACEAE	*Nepenthes madagascariensis* Poir.	Kapilanomba	Entire plantEau dans l’urne	Adhesion of placental-malaria-Albumin - Filariasis - Gonorrhea Syphilis-ear disease	Endemic to Madagascar
NYMPHEACEAE	*Nymphaea nouchali* Burm. f.	Tatamo	Tubers	Hemorrhoids - Pelvic Pain	Comoros, Mascarenes, Africa, Asia
OLACACEAE	*Olax emirnensis* Baker	Soazanahary	Leaf	Placental apposition - Malaria - Hepatitis - Epilepsy - Self-defense against witchcraft - Dysentery - Fatigue - Medicinal plant magic	Endemic to Madagascar
OPHIOGLOSSACEAE	*Ophioglossum* L.	Tsipanga	Leaf	Childbirth	
ORCHIDACEAE	*Angraecum sp.*	Valily	Entire plant	fortifying	
PANDANACEAE	*Pandanus sp*	Vakoana	Leaf	Fatigue - Impotence	
PASSIFLORACEAE	*Passiflora edulis* Sims	Garana	Leaf	Tension - Parasites	naturalized in Madagascar
PHYLLANTHACEAE	*Phyllanthus sp*	Masombero	Leaf	Apposition of the placenta	
PHYSENACEAE	*Physena madagascariensis* Thouars ex Tul.	Resojo	Bark	Sore throat - Anemia - Against poison	Endemic to Madagascar
PIPERACEAE	*Piper nigrum* L.	Poivre	Seeds	Dental disease - Poultice - Joint pain	
PITTOSPORACEAE	*Pittosporum verticillatum* Bojer	Memboloha	Leaf- Bark	Malaria - Adhesion of placental	Endemic to Madagascar
POACEAE	*Cymbopogon citratus* (DC.) Stapf	Veromanitra	Entire plant	Fever	Australasia/Pacific, Africa, Asia, New World
POACEAE	*Cynodon dactylon* (L.) Pers.	Kindresy	Entire plant	Albumin - Malaria - Liver Disease - Menstrual Pain - Laxative	Australasia, Africa, New World
POACEAE	*Eleusine indica* (L.) Gaertn.	Tsipihipihina	Entire plant	Stomach pain	Africa, Asia, New World
POACEAE	*Hyparrhenia rufa* (Nees) Stapf	Verofehana	Entire plant	Epilepsy - Cracks skin of the feet	Africa, Asia, New World
POACEAE	*Imperata cylindrica* (L.) Raeusch.	Tenina	Leaf	Intoxication - Gonorrhea - Pneumonia - Tonsillitis - Measles - Tension	Naturalized in Madagascar
POACEAE	*Oryza sativa L.*	Vary	Bud	birth	cultivated in Madagascar
POACEAE	*Panicum maximum* Jacq.	Ahitry	Leafs	Wounds	Madagascar, Africa, New World
POACEAE	*Sporobolus africanus* (Poir.) Robyns & Tournay	Ahitry	Entire plant	Allergy	Australasia, Africa, New World
POACEAE	*Zea mays* L.	Katsaka	Barbe	gonorrhea	cultivated in Madagascar
POLYGONACEAE	*Persicaria senegalensis* (Meisn.) Soják	Fotsimbarinako	Root	Malaria	Naturalized in Madagascar
RUBIACEAE	*Canthium sp.*	Fotsikahitry	Leaf	Epilepsy	
RUBIACEAE	*Coffea sp.*	Kafe	Leaf	Malaria	
RUBIACEAE	*Danais cernua* Baker	Fangalalemy	Leaf Bark	Syphilis - Tooth Decay	Endemic to Madagascar
RUBIACEAE	*Paederia foetida* L.	Ahimembo	Leaf	Evacuation of the placenta - Headaches	
RUBIACEAE	*Psychotria sp*	Sariloa	Leaf	diarrhea	
RUTACEAE	*Cedrelopsis grevei* Baill.	Hafatraina	Leaf Bark	Stomachaches - Acne	Endemic to Madagascar
RUTACEAE	*Citrus aurantium* L.	Voasary makirana	Fruit	Cough - Malaria	Naturalized in Madagascar
RUTACEAE	*Citrus sinensis* (L.) Osbeck	Voangy gasy	Leaf	Evacuation of placenta-Malaria	
SALICACEAE	*Homalium axillare* (Lam.) Benth.	Fotsiakara	Bark	burns	Endemic to Madagascar
SALICACEAE	*Scolopia sp*	Hazofotsy	Bark	Rheumatism	
SAPINDACEAE	*Litchi chinensis* Sonn.	Letchis	Leaf	diarrhea	cultivated in Madagascar
SARCOLAENACEAE	*Leptolaena pauciflora* Baker	Fatra	Bark	Syphilis	Endemic to Madagascar
SARCOLAENACEAE	*Sarcolaena multiflora* Thouars	Hela	Leaf	Evacuation of placenta	Endemic to Madagascar
SCHIZACACEAE	*Lygodium lanceolatum* Desv.	Sofin’akanga	Leaf	Pancrea pain - Gonorrhea - Tension - Evacuation of placenta	Native to Madagascar
SIMARUBACEAE	*Quassia sp.*	Rembiky	Leaf	Aphrodisiac	
SMILACEAE	*Smilax anceps* Willd.	Roindambo	Leaf	Convulsion - Pregnancy - Fatigue - Boil	Comoros, Mascarenes, Africa
SOLANACEAE	*Capsicum annuum* L.	Sakaipilo	Fruit	Rheumatism - Pain	Naturalized in Madagascar
SOLANACEAE	*Datura inoxia* Mill.	Ramiary	Leaf	Asthma - Calming	Naturalized in Madagascar
SOLANACEAE	*Nicotiana tabacum* L.	Paraky	Leaf	Nosebleed	Naturalized in Madagascar
SOLANACEAE	*Solanum erythracanthum* Bojer ex Dunal	Angivy	Fruit	Cough	Endemic to Madagascar
STILBACEAE	*Nuxia capitata* Baker	Valanirana	Leaf	Cough - Tonic - Tapeworm	Endemic to Madagascar
STRELITZIACEAE	*Ravenala madagascariensis* Sonn.	Ravinala	Leaf	Tension	Endemic to Madagascar
TACCACEAE	*Tacca leontopetaloides* (L.) Kuntze	Tavolo	Tuber	Malnutrition	Naturalized in Madagascar
THYMELACEAE	*Gnidia danguyana* Leandri	Avoha	Leaf	Bleeding - Parasites	Endemic to Madagascar
ULMACEAE	*Trema orientalis* (L.) Blume	Andrarezina / Vakoky	Leaf	Evacuation of the placenta - Dental Disease	Africa
VACCINACEAE	*Vaccinium* sp.	Voakaramy	Leaf	Anemia - Diabetes	
ZINGIBERACEAE	*Aframomum angustifolium* (Sonn.) K. Schum.	Longoza	Leaf	Splinter	Africa
ZINGIBERACEAE	*Curcuma longa* L.	Tamotamo	Leaf	Albumin - Pregnancy - Malaria - Jaundice Viral	
ZINGIBERACEAE	*Hedychium coronarium* J. Koenig	Longoza	Leaf	Evacuation of the placenta - Scabies	
ZINGIBERACEAE	*Zingiber officinale* Roscoe	Sakaintany	Tuber - Leaf	Pregnancy: Nausea - Evacuation of placenta-cough-diarrhea	

Complete list of the vernacular names, scientific identification, use and distribution of all the species mentioned during ethnobotanical interviews.

**Figure 2:**
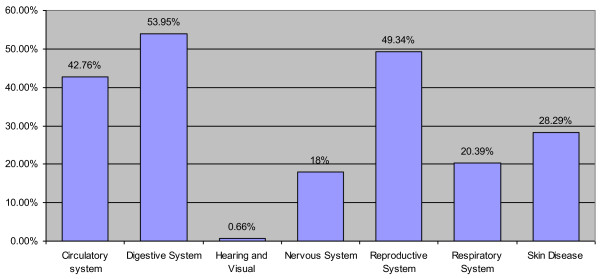
Frequency of diseases mentioned as treated by a medicinal species.

Medicinal plants are mainly used in the care of the digestive system (53.95%), followed by reproductive system (49.34%), then the circulatory system with 42.76%. Then, the plants used against skin diseases have a frequency of use of 28.29%, those used against diseases of the respiratory system with 20.39%. Eighteen percent (18%) of plants are taken for the care of diseases related to nervous systems, those used against diseases associated with hearing and visual are a minority (0.66% only) (Figure 2).

**Figure 3:**
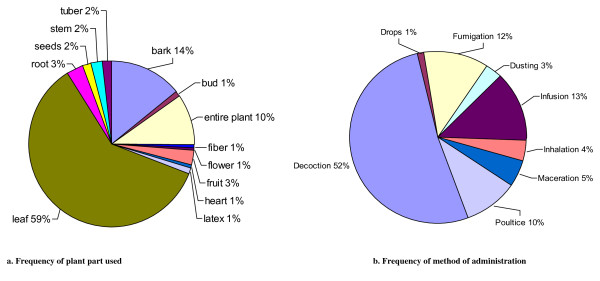
**Distribution of type of plant part used (a.) and method of medicinal administration. (b.)** Percentage of citations for plant parts used and method of administration for medicinal plant treatment as reported during the interviews. Some plants are noted as having multiple medicinal uses with multiple parts of plants utilized.

In the rural commune of Mahabo-Mananivo, leaves are most often cited as the part used for medicinal treatment, followed by bark and entire plant. Decoction is the most used method of preparation with a percentage of 51.60%. It is followed by infusions (13.07%), fumigation (12.40%), poultice (10.45%), maceration (4.58%), inhalation (3.90%), dusting (2.60%) and drops (1.40%) (Figure 3).

Among the medicinal plants collected, a majority are sourced from the littoral forest of Agnalazaha while the cultivated fields, weedy disturbed areas, marshes, savannah, savoka (fallow fields), and river follow up (Table 3).

**Table 3:** Frequency of localities where medicinal plants are sourced near and around Agnalazaha Forest

**Sampled locations**							
	**Forest**	**Marsh**	**Savanna**	**Savoka**	**River**	**Cultivated**	**Disturbed areas**
**Frequency (%)**	40	11	7	4	4	20	14

## Discussion

Our focus on the use of medicinal plants by women of Mahabo-Mananivo reinforced the notion that female caregivers are the first line of health care in many Malagasy homes. We found that traditionally, men collect the medicinal plants while women were mostly responsible for the drying, storage and preparation of the plant to take care of the family members. Reproductive, prenatal and postpartum health were the most frequently cited use for medicinal plants in women’s health, a trend seen worldwide [26], however, the women’s pharmacopeia was not limited to reproductive and childbirth care and many medicinal species from Agnalazaha Forest are used to treat multiple diseases. We found eight native species that were very well known, and were used to treat multiple diseases. *Voacanga thouarsii* is used during childbirth and for the treatment of gonorrhea, syphilis, mycosis, wounds, hypertension and is also used for the care of the digestive tract and stomach ulcers. *Cinnamosma madagascariensis* treats dental decay and general oral care, malaria, and for care of complications after childbirth. *Olax emirnensis* is used during childbirth, and to treat malaria, hepatitis, epilepsy, dysentery, fatigue, and thought to have magical properties and to provide protection against witchcraft. *Syzygium emirnense* is used in childbirth, diarrhea, dental disease, and scabies. *Nepenthes madagascariensis* is used during childbirth, and for treatment of malaria, filariasis, ear infections, syphilis, and gonorrhea. *Phyllarthron madagascariense* is taken to support breastfeeding, to treat malaria and combat fatigue. *Suregada boiviniana* helps to evacuate the placenta and treat epilepsy, dysentery, and malaria. *Asteropeia micraster* also helps to evacuate the placenta and treat diarrhea, fatigue and mumps. Our study found that many of the medicinal species sourced from Agnalazaha Forest were also utilized for other daily living needs. Native medicinal species may also be used as timber, construction materials, and firewood. Conservation concerns mostly lie in the overuse of these valuable daily living species. Conversations with community members highlighted the concern and interest they had for protecting the natural resource of Agnalazaha Forest while ensuring the forest could still be used. It is our goal that through careful ethnobotanical studies of the modern use of Agnalazaha Forest, we can help the community of Mahabo-Mananivo understand their forest use and establish community driven sustainable conservation plans.

## Conclusions

This study highlighted the diversity of medicinal plants used by women and female healers in the Commune of Mahabo-Mananivo. From the perspective of plant diversity, 152 species of medicinal plants in 134 genera and 79 families were identified during the ethnobotanical surveys. First, there is widespread use of medicinal plants that affect the digestive, reproductive and circulatory system. The eight native species widely used are *Cinnamosma madagascariensis*, *Voacanga thouarsii*, *Nepenthes madagascariensis*, *Syzigium emirnense, Olax emirnensis, Phyllarthron madagarascariensis, Suregada boiviana*, and *Asteropeia micraster*. This work is only the beginning of a comprehensive study on the ethnobotany of medicinal plants utilized by the community Mahabo-Mananivo from the Agnalazaha Forest. Further studies encompassing ecophysiological, pharmacological and ecological studies are necessary to build a more complete picture on how these rare and compelling littoral forests are used. By documenting the use littoral forest species, we hope to add to the value of these rare forests but also highlight the importance of biodiversity on the health and wellbeing of a community.

## Competing interests

The authors report no competing interests.

## Authors’ contributions

All authors participated in the design of the study and conducted fieldwork. MR analyzed the data. MR and ARK wrote the manuscript. All authors read and approved the final manuscript.

## Supplementary Material

Additional file 1 **Ethnobotanical questionnaire.** PDF of the questionnaire used during ethnobotanical interviews. Click here for file
