# Supplementary material for: Medicinal plants used by women from Agnalazaha littoral forest (Southeastern Madagascar)
Source: J Ethnobiol Ethnomed. 2013 Nov 4;9:73. doi: 10.1186/1746-4269-9-73 (PMC3827988; doi:10.1186/1746-4269-9-73)
Supplement: Additional file 1 — Ethnobotanical questionnaire. PDF of the questionnaire used during ethnobotanical interviews. [file 1746-4269-9-73-S1.pdf]

ADDITIONAL FILE 1

FICHE D'ENQUETE ETHNOBOTANIQUE

Date :……………………… **N°**………………… **Village** :………………………………..

Enquêteur : RAZAFINDRAIBE Nambinintsoa Mendrika

Personne enquêtée :……………………………………………

Questionnaires

Mettre une croix dans la case que vous estimez convenable.

Prière de repondre de façon précise et honnête.

1. **Age :** A1☐ A2 ☐ A3 ☐ A4 ☐ A5 ☐ A6 ☐
2. **Genre :** Masculin ☐ Féminin ☐
3. **Niveau d'étude** : Analphabète ☐ Primaire ☐ Secondaire ☐ Universitaire ☐
4. **Situation familiale :** Célibataire ☐ Marié(e) ☐ Veuf(ve) ☐
5. **Salaire :** Inférieur à 80 000 Ar ☐ 80 000 Ar ☐ Supérieur à 80 000 Ar ☐
6. **Quand vous vous sentez malade, vous vous adressez :**

   A la médecine traditionnelle ☐ , pourquoi ? efficace ☐ moins chère ☐ acquisition ☐ médicament inéfficace ☐

   A la médecine moderne ☐ , pourquoi ? efficace☐ plus précise ☐ toxicité des plantes ☐

   Si c'est les deux, quelle est la première ? médecine moderne ☐ médecine traditionnelle ☐
7. **Concernant les résultats des soins :**

   Guérison ☐ Amélioration ☐ Intoxication ☐

   Evolution de la maladie ☐ Effets secondaires ☐
8. **Utilisez vous des plantes avec des doses précises :** oui ☐ non ☐
9. **Lorsque vous voulez utilisez une plante, vous vous adressez aux :**

   Expériences des autres ☐ Guérisseurs ☐
10. Connaissez vous des plantes toxiques de la région / utilisées en médecine traditionnelle?

    (Lesquelles ?) ………………………………………………………………………………………

    ……………………………………………………………………………………………………

    ……...

11. **Type de maladie :**

1. Appareil respiratoire 2. Appareil digestif 3. Appareil circulatoire 4. Appareil génital

5. Maladies de la peau 6.Système nerveux 7. Appareil auditif 8. Appareil visuel

12. **Partie utilisée :**

1. Partie souterraine 2. Ecorce 3. Feuilles 4. Bourgeons

5. Fleurs 6. Fruits 7. Plante entière

13. **Mode de préparation :**

1. Décoction 2. Fumigation 3. Infusion 4. Macération 5. Inhalation

6. Poudrage 7. Poudrage 8. Cataplasme 9. Goutte

14. **Lieu de prélèvement :**

1. Forêt 2. Marécage 3.Savane 4. Savoka 5. Rivière 6. Culture 7. Village

15. **Récapitulatif**

Espèce végétale	Type de maladie	Partie utilisée	Mode de préparation	Lieu de prélèvement
